# Defect Detection in Textures through the Use of Entropy as a Means for Automatically Selecting the Wavelet Decomposition Level

**DOI:** 10.3390/s16081178

**Published:** 2016-07-27

**Authors:** Pedro J. Navarro, Carlos Fernández-Isla, Pedro María Alcover, Juan Suardíaz

**Affiliations:** 1 División de Sistemas e Ingeniería Electrónica (DSIE), Universidad Politécnica de Cartagena, Campus Muralla del Mar, s/n, Cartagena E-30202, Spain; pedroj.navarro@upct.es (P.J.N.); carlos.fernandez@upct.es (C.F.-I.); 2 División de Innovación en Sistemas Telemáticos y Tecnología Electrónica (DINTEL), Universidad Politécnica de Cartagena, Campus Muralla del Mar, s/n, Cartagena E-30202, Spain; juan.suardiaz@upct.es

**Keywords:** texture defect detection, wavelet transform, Shannon entropy, automatic band selection

## Abstract

This paper presents a robust method for defect detection in textures, entropy-based automatic selection of the wavelet decomposition level (EADL), based on a wavelet reconstruction scheme, for detecting defects in a wide variety of structural and statistical textures. Two main features are presented. One of the new features is an original use of the normalized absolute function value (NABS) calculated from the wavelet coefficients derived at various different decomposition levels in order to identify textures where the defect can be isolated by eliminating the texture pattern in the first decomposition level. The second is the use of Shannon’s entropy, calculated over detail subimages, for automatic selection of the band for image reconstruction, which, unlike other techniques, such as those based on the co-occurrence matrix or on energy calculation, provides a lower decomposition level, thus avoiding excessive degradation of the image, allowing a more accurate defect segmentation. A metric analysis of the results of the proposed method with nine different thresholding algorithms determined that selecting the appropriate thresholding method is important to achieve optimum performance in defect detection. As a consequence, several different thresholding algorithms depending on the type of texture are proposed.

## 1. Introduction

Defect detection plays a vital role in automatic inspection in most production processes (food, textile, bottling, timber, steel industries, etc.). In many of these processes, quality controls still depend to a large extent on the training of specialized inspectors. Manual inspection involves limitations in terms of accuracy, coherence and efficiency when detecting defects. This is due to the fact that inspectors are prone to suffer fatigue, boredom or simply fail to pay sufficient attention because of the repetitive nature of their tasks [1]. To deal with these problems, human inspectors are being substituted by automatic visual inspection systems [2,3,4].

Texture analysis provides a very powerful tool to detect defects in applications for visual inspection, since textures provide valuable information about the features of different materials.

**Figure 1:**
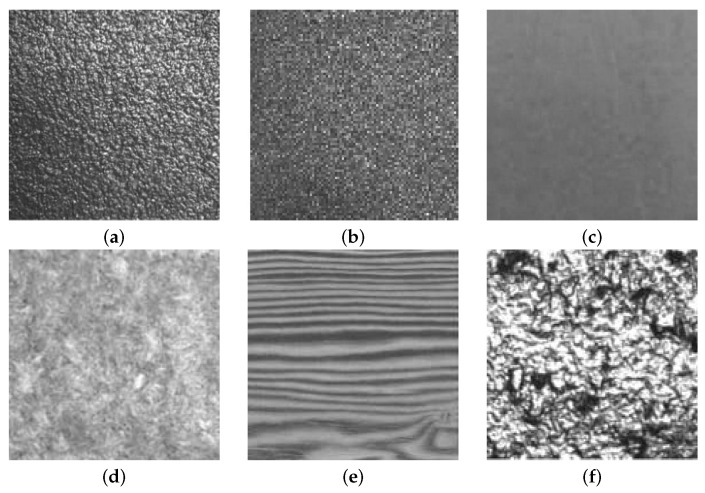
Some statistical textures: (**a**) sandpaper, coarse appearance; (**b**) sandpaper, fine appearance; (**c**) painted surface, fine appearance; (**d**) wool, isotropic; (**e**) wood, high directionality; (**f**) cast metal, irregular.

In computer vision, texture is broadly classified into two main categories: statistical and structural [5]. Textures that are random in nature are well suited for statistical characterization. Statistical textures do not have easily identifiable primitives; however, some visual properties can usually be observed, such as directionality (directional versus isotropic), appearance (coarse versus fine) or regularity (regular versus irregular) (e.g., wool, sand, wood, etc.) (Figure 1).

**Figure 2:**
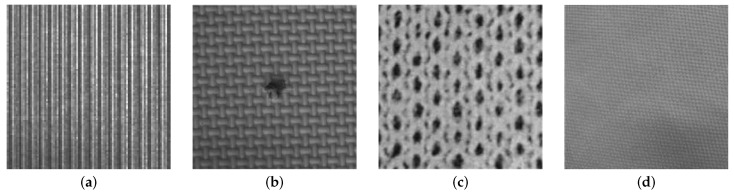
Several examples of structural textures: (**a**) milled surface; (**b**) bamboo weave; (**c**) fabric, coarse appearance (p1); (**d**) fabric, fine appearance (p1).

**Figure 3:**
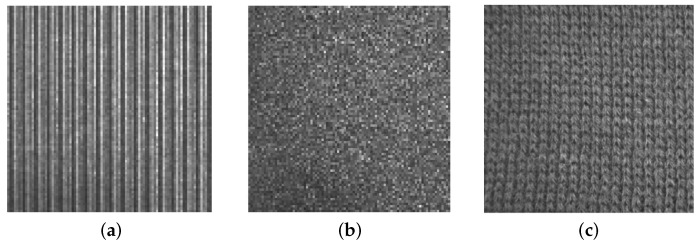
Some homogeneous textures: (**a**) structural texture with directional pattern; (**b**) isotropic statistical texture; (**c**) isotropic patterned texture.

**Figure 4:**
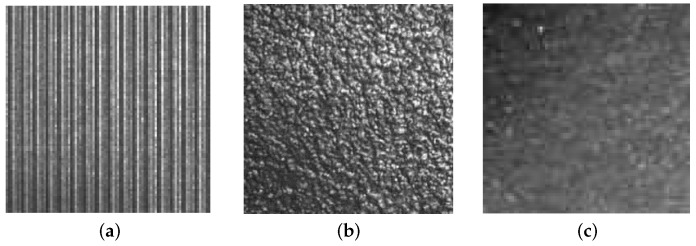
Homogeneous and inhomogeneous textures: (**a**) homogeneous structural texture; (**b**) homogeneous statistical texture; (**c**) inhomogeneous texture.

On the other hand, structural textures, also called patterned textures, are characterized by a set of primitives (texels) and placement rules. The placement rules define the spatial relationships between the texels, and these spatial relationships may be expressed in terms of adjacency, closest distance or periodicities. The texels themselves may be defined by their gray level, shape or homogeneity of some local property (e.g., milled surfaces, fabric, etc.) (Figure 2). Statistical texture patterns are isotropic, while patterned textures can be classified into oriented (directional) or non-oriented (isotropic) [6] (see Figure 3). A homogeneous texture contains repetitive properties everywhere in an image [7]; if repetitive self-similar patterns can be found, it is possible to talk about homogeneous structural texture (Figure 4a). A homogeneous statistical texture cannot be described with texture primitives and displacement rules; the spatial distribution of gray levels is rather stochastic, but the repetition, self-similarity properties still hold (Figure 4b). If there is no repetition or spatial self-similarity, a texture may be defined as inhomogeneous (Figure 4c).

More recently, Ngan [8] provided a new approach, which defines patterned textures in terms of an underlying lattice, composed of one or more motifs, whose symmetry properties are governed by 17 wallpaper groups; wallpaper groups, also known as crystallographic groups, are well defined in mathematic algebra [9]. Figure 2c,d shows two patterned textures classified in the p1 wallpaper group.

In a recent and complete review of defect detection in textures, Xie [10] classified texture analysis techniques in the following categories: statistical techniques [11,12,13], structural techniques [14,15], filter-based techniques [16,17,18] and model-based techniques [19,20,21], while in his review, Kumar [22] sets three categories of defect detection in fabric: the statistical approach, the spectral approach and the model-based approach. Ngan [23] proposes a new classification of techniques for defect detection in textures into motif and non-motif-based approaches. While the traditional (non-motif) techniques can be sub-divided into statistical methods, spectral methods, model-based methods, learning and structural methods (since texture is composed of one main lattice with only one motif), the motive-based techniques [23,24] use the difference and energy variance among different motifs.

Clustering techniques are used in many defect detection methods; these methods are mainly based on the extraction of texture features. Such features are obtained using different techniques, such as co-occurrence matrix [25,26,27,28], Fourier transform [7,29,30,31,32], Gabor transform [33,34,35,36,37] or the wavelet transform [38,39,40,41].

Spectral-approach techniques provide either the frequency contents of a texture image (Fourier transform) or spatial-frequency analysis (Gabor filters, wavelet transform (WT)).

Fourier transform shows good results when applied over texture patterns with high directionality or regularity, because the information about the directionality and periodicity of the texture pattern is well recognizable in the 2D spectrum, although it fails when attempting to determine the spatial localization of such patterns. Concerning spatial localization, Gabor filters provide better accuracy, but they show a lack of reliability when processing natural textures, since there is no single filter resolution that can localize a structure. The main advantage that WT has over the Gabor transform is that it makes it possible to represent the textures in the appropriate scale because of the variation of the spatial resolution it provides.

The suitability of WT for use in image analysis is well established: a representation in terms of the frequency content of local regions over a range of scales provides an ideal framework for the analysis of image features, which in general are of different sizes and can often be characterized by their frequency domain properties [42]. This makes the wavelet transform an attractive option when segmenting textures, as reported by Truchetet [43] in his review of industrial applications of wavelet-based image processing. He reported different uses of wavelet analysis in successful machine vision applications: detecting defects for manufacturing applications for the production of furniture, textiles, integrated circuits, etc., from their wavelet transformation and vector quantization-related properties of the associated wavelet coefficients; the sorting of ceramic tiles and the recognition of metallic paints for car refinishing by combining color and texture information through a multiscale decomposition of each color channel in order to feed a classifier; printing defect identification and classification (applied to printed decoration and tampoprint images) by analyzing the fractal properties of a textured image; image database retrieval algorithms basing texture matching on energy coefficients in the pyramid wavelet transform using Daubechies wavelets; face recognition using the “symlet” wavelet because of its symmetry and regularity; online inspection of a loom under construction using a specific class of the 2D discrete wavelet transform called the multiscale wavelet representation with the objectives of attenuating the background texture and accentuating the defects; online fabric inspection device performing an independent component analysis on a sub-band decomposition provided by a two-level DWT in order to increase the defect detection rate.

### Background and Contributions

Wavelet transform has resulted in two groups of techniques for detecting defects: (1) direct thresholding methods [25,44,45,46,47], whose operation is based on the background attenuation provided by the WT in the successive decomposition levels (such attenuation allows enhancing defects, which can be segmented by utilizing direct thresholding [48]); and (2) methods based on extracting texture features by means of WT [41,42]. After obtaining texture features, feature vectors are formed, and different classifiers are used on them (neural networks, Bayes classifiers, etc.).

In the present paper, a direct thresholding method was decided to be used due to the several disadvantages that methods based on feature vectors present (high computing costs and difficulties to set stop criteria from the proximity-based techniques or training difficulties posed by learning-based techniques). If direct thresholding methods are used, a couple of important challenges have to be faced: (a) how to select the optimal wavelet decomposition level; and (b) determining which bands enable the elimination of the maximum information relative to the texture pattern, which involves two dangerous risks: (a) features of the texture pattern can merge with those of the defects due to an excessive wavelet decomposition; and (b) false positives may appear due to a defective reconstruction scheme.

A review of the literature on texture defect detection methods using wavelet transforms shows a great deal of interest in methods for automatic selection of the optimal wavelet decomposition level due to the potential industrial applications. Of these, the best performance is offered by the methods proposed by [25,46,47], the Tsai and Han methods being the ones that report better results. Tsai proposes an image reconstruction approach based on the analysis and synthesis of wavelet transforms well suited for inspecting surface defects embedded in homogeneous structural and statistical textures. To achieve this, the method removes all regular and repetitive texture patterns from the restored image by selecting proper approximation or detail subimages for wavelet synthesis. In this way, an image where defects are highlighted is obtained. Finally, the method carries out a binarization based on the average and the variance to segment the defects. To find defects in statistical textures with isotropic patterns, the Tsai method uses the approximation image obtained with an optimal resolution level calculated by means of a ratio between two consecutive energy levels. Han reports a method for defect detection in images with high-frequency texture background. Wavelet transform is used to decompose the texture images into approximation, horizontal, vertical and diagonal subimages. This method uses only the approximation subimage, due to the fact that textures are always high-frequency elements of the image. The optimal decomposition level is the maximum variation of the local homogeneity calculated at two successive wavelet decomposition levels. Local homogeneity is one of the 28 features defined by Haralik et al. for texture characterization computed over the co-occurrence matrix [49]. Once the appropriate decomposition level has been selected, Han uses the approximation subimage to reconstruct the image. After that, Otsu’s method is applied to segment the defects. However, as will be seen further in this text, both methods present serious difficulties when processing the large set of textures presented in this paper and when quantifying the size of the defect. This last feature is crucial for many industrial applications: for example in maintenance, tasks where the product is not discarded, but repaired.

This paper proposes a new approach to face the aforementioned inconveniences based on the normalized absolute function value (NABS) and Shannon’s entropy calculation. The main contributions can be summarized as follows:

1. A numeric analysis of the normalized absolute function value (NABS) at different wavelet decomposition levels, which makes it possible to determine the texture directionality. The proposed algorithm makes it possible to classify into high and low directionality patterns, depending on the variation in the slope of the NABS.
2. A novel use of the normalized Shannon’s entropy has been formulated, calculated over different detail subimages, in order to determine the optimal decomposition level in textures with low directionality. For this purpose, it is proposed to calculate this optimal decomposition level as the maximum ratio between the entropy of the approximation subimage and the total entropy, as the sum of entropies calculated for every subimage. This ratio provides better results when detecting defects in a wider range of textures.

This article is organized as follows: Section 1 gives a brief overview of the concerned problem and highlights the paper contribution; also, it examines the background of wavelet decomposition and presents the mathematical notation that will be used throughout the rest of the paper; Section 2 shows a brief description about the wavelet transform; Section 3 presents the proposed method: entropy-based automatic selection of the wavelet decomposition level (EADL); Section 4 presents the results when the developed method was applied to different real textures; finally, Section 5 presents the conclusions.

## 2. Wavelet Decomposition and Mathematical Notation

Wavelet transform can be applied on an image $f\left(x,y\right)$ in 256 gray levels and of size X×Y by means of a convolution (linear, periodic or cyclic), through two filters (a low-pass filter: *L*; and a band-pass filter: *H*), and by taking, from the resulting image, a sample out of two. This process is applied to all the rows, and then, with the resulting rows to all of the columns, provides four subimages defined as LL (*L* filter on rows and columns), LH (*H* filter on rows and *L* filter on columns), HL (*L* filter on rows and *H* filter on columns) and HH (*H* filter on columns and rows). These four subimages share the same origin, and their size is a quarter of the original size. One of them (LL) is referred to as the approximation subimage. It is defined as $f_{LL}^{\left(1\right)}\left(x,y\right)$ and represents an approximation of the original image. The other three subimages are referred to as detail subimages: horizontal detail subimage or $f_{LH}^{\left(1\right)}\left(x,y\right)$; vertical detail subimage or $f_{HL}^{\left(1\right)}\left(x,y\right)$; and diagonal detail subimage or $f_{HH}^{\left(1\right)}\left(x,y\right)$. According to this notation, the original image can be represented in this way: $f\left(x,y\right)=f_{LL}^{\left(0\right)}\left(x,y\right)$.

From this first decomposition level, wavelet transform can be applied again on the previous approximation image, achieving in this way, again, a new approximation image and three detail subimages. $f_{LL}^{\left(j\right)}\left(x,y\right)$ represents the approximation image obtained in the decomposition level *j*. From that image, subimages $f_{LL}^{\left(j+1\right)}\left(x,y\right)$, $f_{LH}^{\left(j+1\right)}\left(x,y\right)$, $f_{HL}^{\left(j+1\right)}\left(x,y\right)$ and $f_{HH}^{\left(j+1\right)}\left(x,y\right)$ can then be obtained. Such subimages form the set of images of the decomposition level $\left(j+1\right)$. In [50], there is a detailed description of the algorithm.

The recovery process of the approximation image $f_{LL}^{\left(j\right)}\left(x,y\right)$, from the four subimages of the decomposition level (j+1), is also described in the basic literature on wavelet transforms. Such a reconstructed image is called *F*:

(1) $$F=W^{\left(-1\right)}\left[f_{LL}^{\left(j\right)}\left(x,y\right)\right]$$

This procedure can be recursively applied until reaching the starting level (j=0).

**Figure 5:**
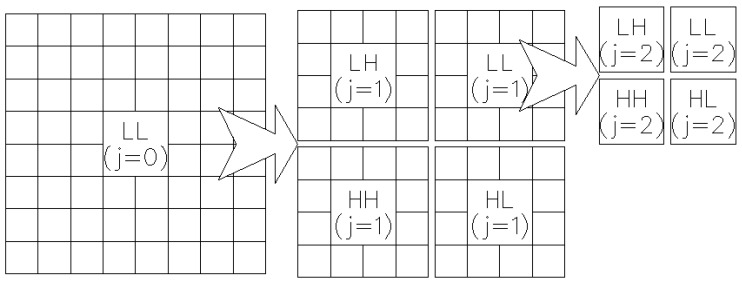
Visual representation of the algorithm of the two-dimension discrete wavelet transform (DWT-2D), where a filtering operation is carried out with a low-pass filter and a band-pass filter in each row. Both filters are applied again on the two resulting matrices, this time in the columns. The result is four subimages, whose size is a quarter of the original image; one of these images is known as the approximation image (double filter LL), and the remaining three are known as detail images: horizontal (LH), vertical (HL) or diagonal (HH).

Figure 5 graphically represents the decomposition process of wavelet transform. Image $f_{LL}^{\left(j\right)}\left(x,y\right)$ is decomposed into the three detail subimages of level (j+1), as well as the new approximation image of level (j+1) that, at the same time, is decomposed into four subimages at level (j+2). Every subimage of level *j* is of size $\left(X/2^{j}\right)\times \left(Y/2^{j}\right)$, where *X* and *Y* represent the dimensions of the original image at level j=0. Thus, a multiresolution analysis of the original image is achieved at different scales, and according to the wavelet signal used in the convolution, it is possible then to find periodic structures, at one scale or another, in a horizontal, vertical or diagonal distribution.

## 3. Entropy-Based Method for Automatic Selection of the Wavelet Decomposition Level

Entropy has been used in many image processing methods: image segmentation [51,52]; thresholding methods [53,54]; Haralick’s texture descriptor, utilized as parameter for gray-level co-occurrence matrices; measurement incorporated with feature vectors [49,55], etc.

A three-stage method for detecting defects in textures is proposed:

1. In order to determine if the texture has high directionality, the NABS value is calculated. If so, the subimage that contains the maximum directionality information is removed, together with the approximation subimage.
2. If the directionality value is low, then the optimum decomposition level is estimated by means of Shannon’s entropy.
3. To determine the optimum thresholding method.

### 3.1. Automatic Detection of High Directionality in Texture

**Figure 6:**
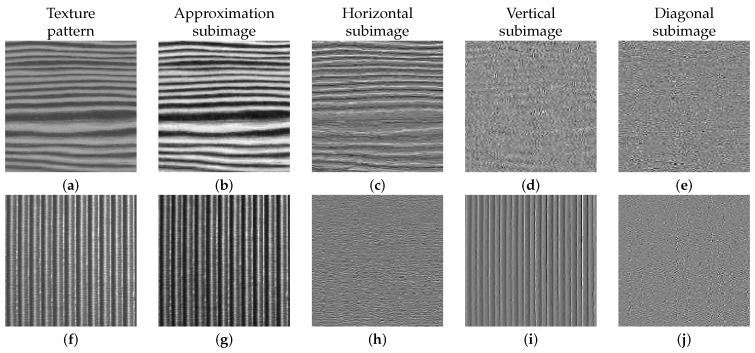
Texture patterns (**a**,**f**) and subimages of the first wavelet decomposition levels for directional textures: aproximation (**b**,**g**); horizontal (**c**,**h**); vertical (**d**,**i**); and diagonal (**e**,**j**).

In high horizontal and vertical directionality patterns, texture information relies to a great extent on the approximation subimage and either in the horizontal or in the vertical subimage, as shown in Figure 6. In these cases, to isolate defects, it is enough to reconstruct the image eliminating the approximation detail (Figure 6b,g) together with the subimage, which contains the maximum information about the pattern directionality in the first decomposition level (Figure 6c,i).

**Figure 7:**
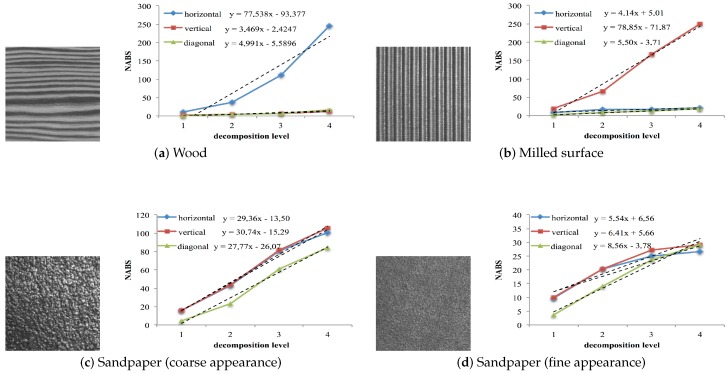
Graphic representation of the normalized absolute function values (NABS) ($NABS_{h}^{j}$, $NABS_{v}^{j}$, $NABS_{d}^{j}$) and their trend line equations calculated at four decomposition levels of images (**a**–**d**). Notice that textures (**a**,**b**) are selected to be highly directional since they show a trend line slope significantly greater than the others corresponding to certain subimages.

In this work, the normalized absolute function value (NABS) was used to separate patterns of structural textures with high directionality from the rest. To determine the directionality of a texture, the values of the NABS of the detail subimages ($f_{LH}^{\left(j)\right)}$, $f_{HL}^{\left(j)\right)}$, $f_{HH}^{\left(j)\right)}$) are calculated at different decomposition levels (see its graphical representation in Figure 7), and the trend line slopes for each of the three sets of values are obtained ($NABS_{h}$, $NABS_{v}$ and $NABS_{d}$). If one of these three slopes is very superior to the other two, it can be considered that the texture has great directionality (h, v or d). Thus, the corresponding subimage (h, v or d) together with the approximation subimage of the first decomposition level are eliminated. The approximation subimage is eliminated in the reconstruction process because it constitutes a rough representation of the original image and, therefore, contains a great deal of information about the pattern’s directionality [46].

The normalized expressions of the absolute value (NABS) of the horizontal, vertical and diagonal detail subimages in level *j* are given by Equations (2)–(4):

(2) $$NABS_{h}^{j}=\frac{1}{N_{pixels}^{j}}\cdot \sum_{x}\sum_{y}\left|f_{LH}^{\left(j\right)}\left(x,y\right))\right|$$

(3) $$NABS_{v}^{j}=\frac{1}{N_{pixels}^{j}}\cdot \sum_{x}\sum_{y}\left|f_{HL}^{\left(j\right)}\left(x,y\right))\right|$$

(4) $$NABS_{d}^{j}=\frac{1}{N_{pixels}^{j}}\cdot \sum_{x}\sum_{y}\left|f_{HH}^{\left(j\right)}\left(x,y\right))\right|$$

for j=1,2,...,J (where $J=\log_{2}N$). $N_{pixels}^{j}=\frac{X}{2^{j}}\times \frac{Y}{2^{j}}$ is the number of pixels at each decomposition level *j*.

Figure 7 shows representative images of statistical (Figure 7a,c,d) and structural (Figure 7b) textures. The first two images represent a pattern with high horizontal and vertical directionality respectively, while the other two images present a statistical texture with a coarse-to-fine appearance.

The graphs to right of each image show the NABS values of the horizontal, vertical and diagonal details ($NABS_{h}^{j}$, $NABS_{v}^{j}$, $NABS_{d}^{j}$) as a function of the decomposition level j=1,2,3,4. Analysis of the graphs shows considerable differences between the NABS values for the high directionality texture patterns and for no directional patterns. This difference is very noticeable in each of the trend line equation slopes, which were obtained using the least squares approach.

It can therefore be concluded that a pattern presents high horizontal, vertical or diagonal directionality if the first-order trend line equation coefficients ($a_{\left\{h,v,d\right\}}$) show variations greater than a given percentage of the maximum of the coefficients.

To simplify the programming, a scale change on the ratio between each of the coefficients and the maximum is applied, expressed as a percentage (Equation (5)), so that the maximum slope value for the three trend lines is obtained when the numerical value $R_{\left\{h,v,d\right\}}$ is zero. Based on the empirical results, a texture may be considered highly directional (horizontal, vertical or diagonal) if the two values $R_{\left\{h,v,d\right\}}$ different from zero are higher than 70%.

(5) $$R_{\left\{h,v,d\right\}}=100\cdot \left(1-\frac{a_{\left\{h,v,d\right\}}}{a_{\left\{h,v,d\right\}}^{max}}\right)$$

Table 1 shows the significant difference between the vertical ($R_{v}$, 95.4%) and diagonal ($R_{d}$, 93.5%) details of the image in Figure 7a and the horizontal detail ($R_{h}$, 0.0%). The table also shows the variation of the horizontal detail ($R_{h}$, 94.7%) and diagonal detail ($R_{d}$, 93.0%) with respect to the vertical detail ($R_{v}$, 0.0%), of the image in Figure 7b. The images in Figure 7a,b can therefore be said to show a high degree of horizontal and vertical directionality, respectively.

The resulting image *F* will be derived from the composition of the rest of detail subimages. In the case of the textures in Figure 7a,b, the new images will be derived through the following expressions:

$$F=W^{-1}\left[f_{HL}^{\left(1\right)}+f_{HH}^{\left(1\right)}\right]$$

$$F=W^{-1}\left[f_{LH}^{\left(1\right)}+f_{HH}^{\left(1\right)}\right]$$

As regarding the behavior of the first-order coefficients of the trend line equation on texture patterns shown in Figure 7a–d, it can be noticed that in the statistical and structural textures with high directionality, there are variations greater than 90% in the trend line equation of the horizontal and diagonal details with respect to the other detail subimages (Figure 7a,b. Therefore, as indicated above, a texture may be considered highly directional if there are two values $R_{\left\{h,v,d\right\}}$ nonzero and higher than 70%.

### 3.2. Automatic Selection of the Appropriate Decomposition Level Using Shannon’s Entropy

In the process described in Section 3.1, it is proven that, at certain decomposition levels, image textures enjoying high directionality are clearly detected and, at the same time, the defects on them. It is very useful to count on algorithms that automatically determine the optimal decomposition level, in which textures and defects are more easily detected. An algorithm based on Shannon’s entropy has been developed, and it is presented below.

**Figure 8:**
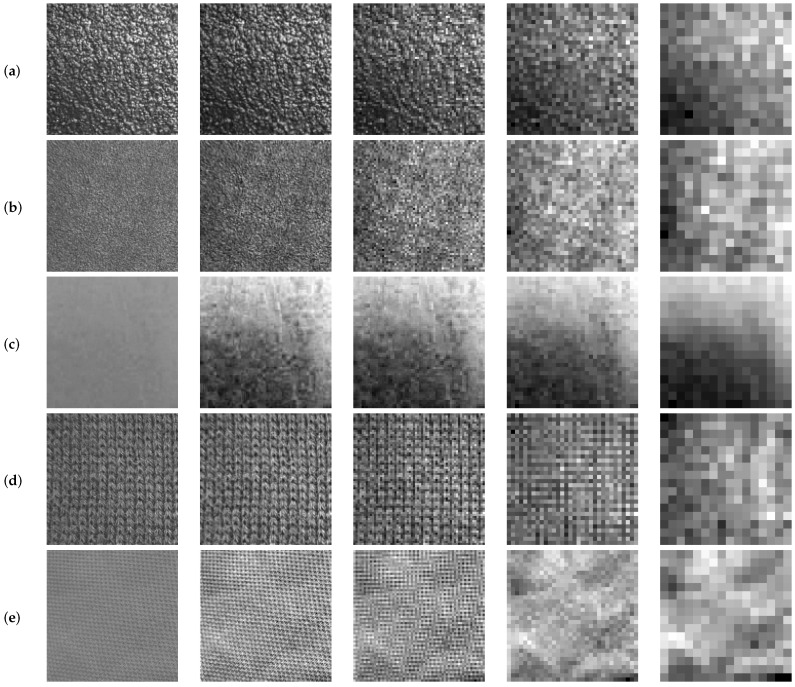
Approximation subimages ($f_{LL}^{\left(j\right)}$) of four wavelet decomposition levels for different textures. (**a**) Sandpaper (course appearance); (**b**) sandpaper (fine appearance) and (**c**) painted-surface: statistical textures; (**d**) fabric (course appearance) and (**e**) fabric (fine appearance): structural textures.

It is already known that Shannon’s entropy describes the level of randomness or uncertainty in an image, i.e., how much information such an image provides. It is possible to state that the higher the value of the entropy, the greater the image quality [56]. Figure 8 shows how texture patterns decrease as the decomposition level of the image is increased. Image degradation level can be measured and quantified at successive levels through Shannon’s entropy.

The entropy value [57,58] is calculated according to the following Equation (6):

(6) $$S\left(X\right)=-\sum_{i=1}^{T}p\left(x_{i}\right)\cdot \log p\left(x_{i}\right)$$

where $X=\left\{x_{1},x_{2},\ldots x_{T}\right\}$ is the set of *T* values on which the entropy function is applied and where the function $p\left(x_{i}\right)$ calculates the probability of occurrence associated with the value $x_{i}$; as our analysis image set *X* (256 gray-level images) consists of these 256 possible values and the probability $p\left(x_{i}\right)$ will derive from the total number of pixels in the image that have, indeed, the gray level $x_{i}$, divided by the total number of pixels in the image (also referred to as $N_{t}$).

In performing the convolution process of the image with a specific wavelet signal, unbounded real values, positive and negative ones, are obtained. Thus, in order to calculate Shannon’s entropy function in each subimage and for each decomposition level *j*, the values of each subimage are firstly transformed over the range of integer values between zero and 255. Therefore, in addition to allowing the visualization of the resulting subimages, entropy can be calculated, as well. For each decomposition level, Shannon’s entropy provides four values: $S_{LL}^{j}$, $S_{LH}^{j}$, $S_{HL}^{j}$ and $S_{HH}^{j}$. Each of these normalized values will be divided by the total number of pixels the subimage has in the decomposition level *j*. Such values are referred to as $S_{s}^{j}$ or $S_{LL}^{j}$ for the entropy of the approximation image and as $S_{h}^{j}$, $S_{v}^{j}$ and $S_{d}^{j}$ (or, respectively, $S_{LH}^{j}$ ,$S_{HL}^{j}$ and $S_{HH}^{j}$) for the entropy values of the horizontal detail, vertical detail and diagonal detail subimages.

The function value of Shannon’s entropy indicates how much information on the texture from the original image remains in each subimage. This function value is calculated on each subimage derived from each decomposition level. According to Equation (6), it can be stated that entropy provides a measure of the histogram: the higher the entropy, the greater the uniformity of the histogram, i.e., the greater the information that the image contains on the texture. As the decomposition level increases, subimages lose information about the texture patterns.

In an optimal decomposition level, it would be possible to eliminate the texture pattern with no significant loss of information on the defects. In order to determine the optimal decomposition level, a ratio value $r_{j}$ will be used, calculated between the entropy value of the approximation subimage and the sum of the four subimages:

(7) $$r_{j}=\frac{S_{s}^{j}}{S_{s}^{j}+S_{h}^{j}+S_{v}^{j}+S_{d}^{j}}$$

Variations of this ratio allow detecting changes in the amount of texture information between two consecutive decomposition levels. The goal is to find the decomposition level causing the maximum variation of the value expressed in Equation (7) with regard to the value in the previous decomposition level. This would indicate that the texture pattern is still present at level *j* and that it disappears at level (j+1), where, however, information on the defects would remain.

For this calculation process, the coefficient ${ADR}_{j}$ is defined as the difference between two values of $r_{j}$ corresponding to two consecutive decomposition levels.

(8) $$ADR_{j}=\left\{\begin{array}{cc} 0 & j=1\\ \left|r_{j}-r_{j-1}\right| & j=2,...,J \end{array}\right.$$

The optimal decomposition level ($J^{*}$) is defined as the level in which $ADR_{j}$ takes the highest value.

(9) $$J^{*}=\arg\left\{\underset{j}{max}\left\{ADR_{j}\right\}\right\}$$

From that level, the decomposition process may end. Beyond that value (for values $j>J^{*}$), it is possible to assume that the approximation image remains sufficiently smoothed: most of the texture patterns have been eliminated, and from now on, going ahead with the decomposition would lead to a loss of information on the defects.

In Table 2, the values of Shannon’s entropy are gathered. They were calculated for the images in Figure 8. The values of the coefficients $r_{j}$, calculated for each image and in each decomposition level, were also gathered, as well as the calculated values of $ADR_{j}$.

Once the optimal decomposition level is determined, the process ends with the reconstruction of the image according to Equation (10).

(10) $$F\left(x,y\right)=W^{-1}\left[f_{LL}^{\left(j\right)}\left(x,y\right)\right]$$

### 3.3. Optimum Thresholding Method

The wavelet-based methods of texture defect detection reviewed here conclude with a thresholding stage. Most authors use recognized methods, such as [59], or methods based on empirical adjustment [46]. These methods do not specify the criteria for the selection of the thresholding technique, and they do not provide objective calculations on the performance of the method. Sezgin’s survey [48] reviews a large number of thresholding methods for defect detection. Sezgin categorizes the thresholding methods into six groups according to the information they use. These categories are:

- histogram shape-based methods,
- clustering-based methods,
- entropy-based methods,
- object attribute-based methods,
- spatial methods and
- local methods.

Sezgin’s findings show that the best thresholding results were achieved with the three first groups of methods.

Several thresholding methods in the final stage of the EADL method have been used to determine the following:

1. How does the selection of thresholding methods influence the final performance of the algorithm?
2. Which is the most suitable thresholding method for segmenting defects according to texture type?

To answer the above questions, the following thresholding methods were selected, attending to the three first groups proposed by Sezgin:

- Histogram shape-based methods: thresholding methods based on gray level average (Ave) and the thresholding method based on computing the minima of the maxima of the histogram (MiMa) [60].
- Clustering-based methods: Ridler’s method [61], Trussell’s method [62], Otsu’s method [59] and Kittle’s method [63].
- Entropy-based methods: Pun’s method [64], Kapur’s method [52] and Johanssen’s method [51].

In order to perform a quantitative analysis of the segmentation method proposed in this paper, the adequate metrics are necessary to be selected. From the set of metrics proposed by Sezgin [48] and Zhang [65,66], the misclassification error (ME) has been selected. ME represents a measurement of the number of misclassified pixels; ME occurs if the foreground (defect) is identified as the background (texture pattern), or vice versa).

(11) $$ME=\frac{\left|BP\cap BT\right|+\left|OP\cap OT\right|}{\left|BP\right|+\left|OP\right|}$$

*ME* is calculated as the relation between the pixels of the test image (segmented by means of the method proposed in this paper) and those of a pattern image (manually segmented). Equation (11) is used to calculate it. BT (background test) and OT (object test) respectively indicate the number of pixels of the texture pattern image and those of the defect in the test image. BP (background pattern) and OP (object pattern) respectively indicate the number of pixels of the texture pattern and those of the defect in the pattern image. The equation proves that the more both images look alike, the smaller ME will be, being zero in the case of a perfect match between the manual segmentation and the automatic segmentation. This indicates, therefore, a maximum efficiency of the method proposed in the present paper.

Equation (12) is used to assess the yield of the segmentation method.

(12) η=100·(1−ME)

**Figure 9:**
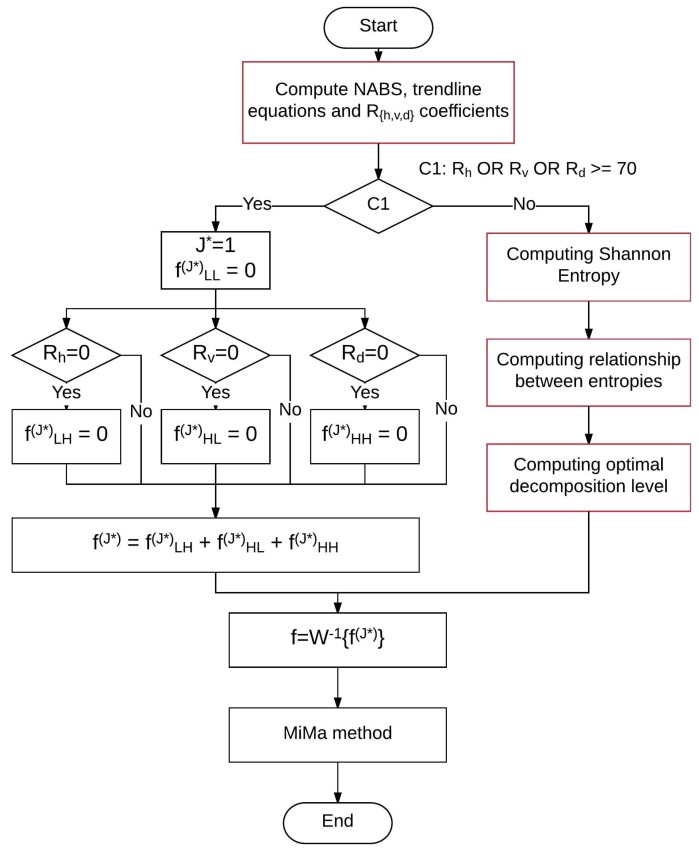
Flowchart to implement the entropy-based automatic selection of the wavelet decomposition level (EADL) method. See Equation (5) for $R_{h}$, $R_{v}$, and $R_{d}$ definition.

Figure 9 contains the flowchart that summarizes the EADL method. The most expensive computational processes are those colored red in the shown flowchart. The computational complexity of each one is:

- Compute NABS: $T\left(n\right)=10\times n^{2}+10\times n+12\equiv \Theta \left(n^{2}\right)$.
- Shannon entropy: $T\left(n\right)=8\times n^{2}+10\times n+k$, where k≈200,000; for values n≥180: $\Theta \left(n^{2}\right)$.
- Compute $r_{j}$ and $J^{*}$: $\Theta \left(\log_{2}n\right)$.

From above individual computational complexities, a total computational complexity of order $\Theta \left(n^{2}\right)$ is derived. On the other hand, the computational complexity of the algorithm used for wavelet decomposition is $90\times n^{3}+\alpha \times n^{2}+33\times n+47+\beta$, being α=212.5 in the most favorable case and α=219.5 in the worst case; the value of *β* indicates the number of instructions executed by function *MbufPut2d()* of the Matrox Imagin Library (MIL). Therefore, the computational complexity of the EADL method is $\Theta \left(n^{3}\times \log_{2}n\right)$. From these calculations, it can be concluded that EADL does not increase the complexity of the process for calculating the Wavelet transforms.

## 4. Results

The EADL method, whose algorithm is shown in Figure 9, was implemented using the C programming language. The EADL method, the automatic band selection method [46] and the adaptive level-selecting method [25] for wavelet reconstruction were tested over a set of 223 images of texture: 115 structural textures: milled surface (29), fabric (67) and bamboo weave (19); and 108 statistical textures: sandpaper surface (29), wood surface (19), wool surface (19), painted surface (21) and cast metal (20).

After checking different mother wavelets (Haar, symlets, biorthonormal, Meyer and coiflets), the Haar-based function with two coefficients has been used as the mother wavelet, because it shows the best ratio between yield and computational cost. The Haar wavelet has been applied until the fourth decomposition level. Higher decomposition levels have proven to lead to the merger of the defects with the texture pattern, which prevents their segmentation.

### 4.1. Statistical Textures

**Figure 10:**
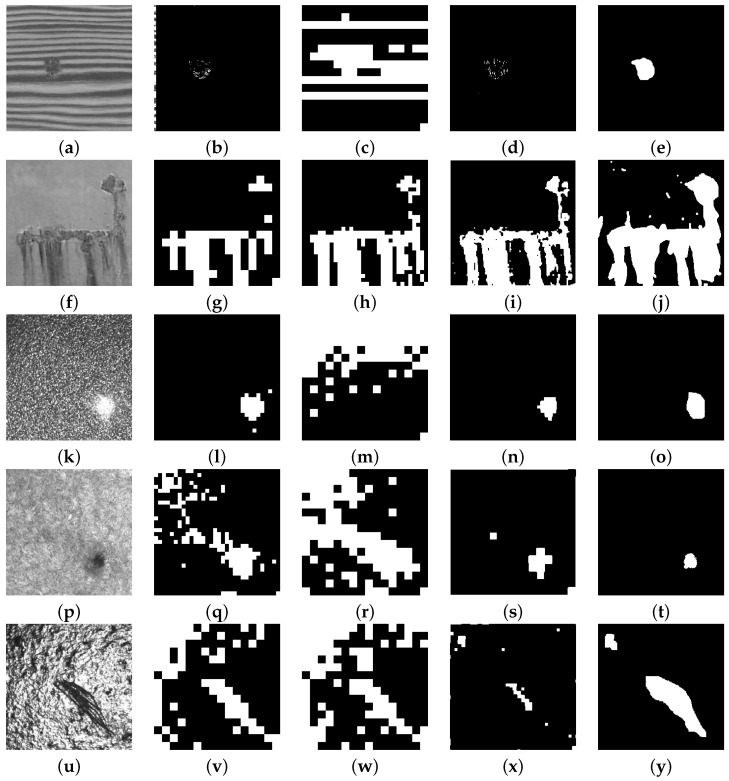
Defects in different statistical textures: (**a**) wood, (**f**) painted surface, (**k**) sandpaper, (**p**) wool, (**u**) cast metal; (**b**,**g**,**l**,**q**,**v**) are the images resulting from the Tsai method; (**c**,**h**,**m**,**r**,**w**) are the images resulting from the Han method; (**d**,**i**,**n**,**s**,**x**) are the images resulting from the EADL method; (**e**,**j**,**o**,**t**,**y**) are the pattern images resulting from segmentation carried out by human inspectors.

Figure 10 shows a representative set of different types of defects in statistical textures obtained from the group of 108 images used to test the EADL method: wood (a), painted surfaces (j), sandpaper (k), wool (p) and cast metal (u).

Table 3 shows the value $r_{j}$ calculated from the Shannon entropy ratio at different decomposition levels for the images of Figure 10 classified as statistical textures; it also shows the optimal decomposition level calculated by means of the EADL method, the automatic band selection method and the adaptive level-selecting method, respectively. Image (a) does not show the $r_{j}$ and $ADR_{j}$ figures since the optimal decomposition level was found with the NABS algorithm ($J^{*}=1$).

Metric analysis (Table 4) showed that if only one thresholding method is used in EADL programming for the group of statistical textures considered, that method should be MiMa, since it was the one that offered the best average performance in defect detection (95.00%). The maximum performance for each type of texture is underlined. On the other hand, if the thresholding method with the best performance is used according to the kind of texture—wood, sandpaper, wool, painted surface or cast metal—two thresholding methods should be used:

- The Kapur method for natural directional textures wood (98.38%), sandpaper (98.47%) and wool (96.75%).
- The MiMa method for artificial irregular isotropic statistical textures painted surface (92.22%) and cast metal (92.25%).

### 4.2. Structural textures

**Figure 11:**
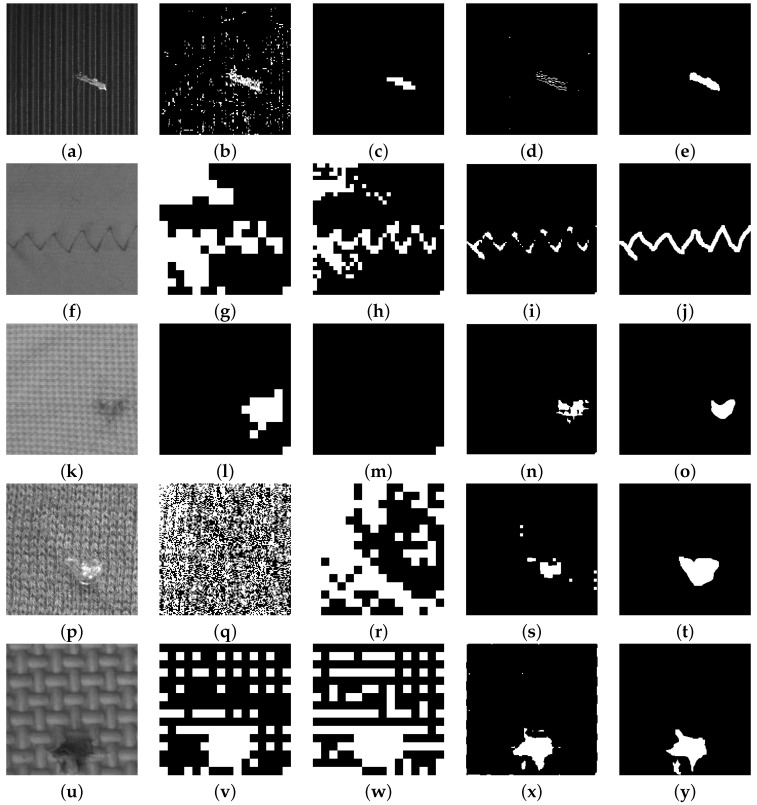
Defects in different structural texture. (**a**) milled surface, (**f**) fabric fine-appearance, (**k**) fabric medium-appearance, (**p**) fabric coarse-appearance and (**u**) bamboo weave; (**b**,**g**,**l**,**q**,**v**) are the images resulting from the Tsai method; (**c**,**h**,**m**,**r**,**w**) are the images resulting from the Han method; (**d**,**i**,**n**,**s**,**x**) are the images resulting from the EADL method; (**e**,**j**,**o**,**t**,**y**) are the ground-truth images resulting from segmentation carried out by human inspectors.

Figure 11 shows five types of defects in patterns of structural texture obtained from a group of 108 images used to test the EADL method. Figure 11 shows: (a) milled surface, (f) fabric fine-appearance, (k) fabric medium-appearance, (p) fabric coarse-appearance and (u) bamboo weave.

Table 5 shows the value $r_{j}$ calculated from the Shannon entropy ratio at different decomposition levels for the images of Figure 11 classified as structural textures. It also shows the optimal decomposition level calculated by means of the EADL method, the automatic band selection method and the adaptive level-selecting method, respectively. Image (a) does not show the $r_{j}$ and $ADR_{j}$ figures since the optimal decomposition level was found with the NABS algorithm ($J^{*}=1$).

Table 6 depicts the numeric values obtained for the thresholding algorithms selected: a value of 100% indicates that the defect detected by EADL fully matches with the same defect when segmented by a qualified human inspector. The maximum performance for each type of texture is underlined.

As in the previous Section 4.1, the efficiency of the EADL method is determined as compared to the use of a single or multiple thresholding methods. The metric analysis results (Table 6) show that MiMa achieved the best average performance (92.22%). However, if the best-performing thresholding method according to the type of texture—milled surfaces, bamboo weave and fabric—needs to be selected, it is best to select:

- Kapur’s method for artificial directional structural textures milled surfaces (99.25%) and bamboo weave (91.11%).
- The MiMa method for natural isotropic structural textures fabric appearance fine (96.25%) and fabric appearance course (86.82%).

Table 7 shows the yields obtained in defect detection with the Tsai, Ngan and EADL methods on 115 structural and 108 statistical texture images. Table 7 also shows the optimal levels of wavelet decomposition ($J^{*}$) obtained by each method. As can be seen, the EADL method performance is higher for the two groups of analyzed textures. It can also be seen that EADL method provides a lower average level of decomposition than the other methods discussed. A lower decomposition level means less degradation of the original image and, therefore, greater detail in detecting defects.

## 5. Conclusions

This paper presents a robust method for detecting defects in a wide variety of structural and statistical textures. An image reconstruction scheme based on the automatic selection of (1) the band, using NABS and (2) the optimal wavelet transform resolution level, using Shannon’s entropy, has been used.

Valuable information about the directionality of the texture patterns can be extracted from the analysis of the NABS value of the horizontal, vertical and diagonal details at different decomposition levels.

A correct wavelet reconstruction scheme has been implemented to remove the texture patterns and highlight the defects in the resulting images.

It is demonstrated that the optimal decomposition levels computed from the Shannon entropy are lower than the ones provided by other methods based on the co-occurrence matrix (Han method) or on energy calculation (Tsai method). This fact implies an increment of information in the image resulting from the wavelet reconstruction scheme. This characteristic, together with the optimal selection of a thresholding method (MiMa), has allowed the EADL method to achieve high performances in defect detection: a 95.00% in statistical textures and a 92.22% in structural textures.

An analysis of the results of the EADL method with nine different thresholding algorithms showed that selecting the appropriate thresholding method is important for achieving optimum performance in defect detection. On the basis of a metric analysis of 223 images, the most appropriate thresholding algorithm for each texture is proposed. The MiMa method proved to be the most appropriate for the textures, such as painted surfaces, cast metal and fabric. However, the Kapur method has been demonstrated to be better with wood, sandpaper surface, wool, milled surface and bamboo weave.

## Figures and Tables

**Table 1:** Trend line equation coefficients and maximum variation ratios of the images in Figure 7.

	1st Order Coefficients *y = ax + b*			Maximum Variation Ratio		
Image	$a_{h}$	$a_{v}$	$a_{d}$	$R_{h}$	$R_{v}$	$R_{d}$
Figure 7a Wood	77.6	3.5	5.0	0%	95.4%	93.5%
Figure 7b milled surface	4.2	78.9	5.6	94.7%	0%	93.0%
Figure 7c sandpaper (coarse)	29.4	30.8	27.8	4.5%	0%	9.7%
Figure 7d sandpaper (fine)	5.6	6.5	8.6	34.9%	24.8%	0%

**Table 2:** Normalized entropies of four decomposition levels for statistical and structural isotropic textures of Figure 8. The optimal resolution level determination ($J^{*}$) is highlighted for every texture.

Decomposition Level	$S_{s}^{j}$	$S_{h}^{j}$	$S_{v}^{j}$	$S_{d}^{j}$	$r_{j}$	${\mathrm{ADR}}_{j}$
Sandpaper-coarse appearance						
j=1	0.0001337	0.0001237	0.0001210	0.0001020	0.2782	-
j=2	0.0005385	0.0005169	0.0005102	0.0004868	0.2624	0.0158
j=3	0.0017080	0.0020344	0.0020738	0.0020759	0.2164	0.0460
$j=4(J^{*})$	0.0080432	0.0072046	0.0071588	0.0068548	0.2749	0.0585
Sandpaper-fine appearance						
j=1	0.0001255	0.0001228	0.0001231	0.0000972	0.2678	-
j=2	0.0005295	0.0005121	0.0005184	0.0005024	0.2568	0.0111
j=3	0.0013122	0.0017993	0.0018528	0.0020630	0.1867	0.0700
$j=4(J^{*})$	0.0069050	0.0058039	0.0060676	0.0059565	0.2792	0.0925
Painted-surface						
j=1	0.0001273	0.0000464	0.0000485	0.0000295	0.5060	-
$j=2(J^{*})$	0.0005614	0.0003787	0.0003889	0.0002652	0.3522	0.1538
j=3	0.0021846	0.0017481	0.0018008	0.0016123	0.2974	0.0548
j=4	0.0083969	0.0076082	0.0076374	0.0066278	0.2774	0.0200
Fabric-coarse appearance						
j=1	0.0001309	0.0001189	0.0001249	0.0000966	0.2778	-
j=2	0.0005388	0.0005157	0.0005202	0.0005054	0.2590	0.0187
$j=3(J^{*})$	0.0014766	0.0018525	0.0019925	0.0021241	0.1983	0.0607
j=4	0.0068620	0.0068641	0.0068436	0.0063836	0.2546	0.0563
Fabric-fine appearance						
j=1	0.0001309	0.0001078	0.0001066	0.0000894	0.3012	-
$j=2(J^{*})$	0.0005310	0.0005461	0.0005289	0.0005220	0.2495	0.0517
j=3	0.0020043	0.0022291	0.0022342	0.0021072	0.2337	0.0158
j=4	0.0080267	0.0078364	0.0078882	0.0079990	0.2528	0.0191

**Table 3:** $J^{*}$ calculated from statistical textures of Figure 10a,f,k,p,u with the EADL method ($r_{j}$ coefficients are shown), with the automatic band selection method (Tsai [46]) and the adaptive level-selecting method (Han [25]). (For $r_{j}$, $ADR_{j}$ and $J^{*}$, see Equations (7)–(9)).

Image in Figure 10		j=1	j=2	j=3	j=4	$J^{*}$		
						EADL	Tsai	Han
(a) wood	$r_{j}$	-	-	-	-	1	1	4
	$ADR_{j}$	-	-	-	-			
(f) painted surface	$r_{j}$	0.3550	0.3020	0.2954	0.2696	2	4	3
	$ADR_{j}$	-	0.0530	0.0067	0.0258			
(k) sandpaper	$r_{j}$	0.3550	0.3020	0.2954	0.2696	3	4	4
	$ADR_{j}$	-	0.0147	0.0311	0.0225			
(p) wool	$r_{j}$	0.2700	0.2469	0.2171	0.2548	4	3	4
	$ADR_{j}$	-	0.0231	0.0298	0.0377			
(u) cast metal	$r_{j}$	0.2765	0.2687	0.2540	0.2529	3	4	4
	$ADR_{j}$	-	0.0078	0.0148	0.0011			

**Table 4:** Numerical values obtained using different thresholding methods on statistical textures: (1) the average method, (2) the minima of the maxima of the histogram (MiMa) method, (3) the Riddler method, (4) the Thrussel method, (5) the Otsu method, (6) the Pun method, (7) the Kapur method,(8) the Johanssen method and (9) the Kittler Method.

Statistical Textures	*N*	(1)	(2)	(3)	(4)	(5)	(6)	(7)	(8)	(9)
Wood	19	91.86	97.81	97.45	94.64	93.57	63.60	98.38	97.21	63.62
Sandpaper surface	29	56.48	98.39	54.72	73.24	78.96	55.00	98.47	91.34	65.31
Wool	19	56.16	94.34	55.12	55.96	73.51	52.23	96.75	87.47	41.21
Painted surface	21	61.26	92.22	71.84	73.97	83.94	59.35	91.12	91.77	86.38
Cast metal	20	58.14	92.25	63.65	62.57	59.60	57.42	62.50	57.54	65.28
Averages	108	64.78	95.00	68.56	72.08	77.91	57.52	89.44	85.07	64.36

N: number of the images.

**Table 5:** $J^{*}$ calculated from structural textures of Figure 11a,f,k,p,u with the EADL method ($r_{j}$ coefficients are shown), with the automatic band selection method (Tsai [46]) and the adaptive level-selecting method (Han [25]) (For $r_{j}$, $ADR_{j}$ and $J^{*}$, see Equations (7), (8), and (9)).

Image in Figure 11		j=1	j=2	j=3	j=4	$J^{*}$		
						EADL	Tsai	Han
(a) Milled surface	$r_{j}$	-	-	-	-	1	1	3
	$ADR_{j}$	-	-	-	-			
(f) Fabric fine-appearance	$r_{j}$	0.3962	0.2872	0.2745	0.2741	2	4	3
	$ADR_{j}$	-	0.1090	0.0127	0.0005			
(k) Fabric medium-appearance	$r_{j}$	0.4855	0.2578	0.2320	0.2370	2	4	4
	$ADR_{j}$	-	0.2278	0.0258	0.0050			
(p) Fabric coarse-appearance	$r_{j}$	0.2669	0.2463	0.2069	0.2349	3	1	4
	$ADR_{j}$	-	0.0206	0.0394	0.0280			
(u) Bamboo weave	$r_{j}$	0.3908	0.2855	0.2580	0.2426	2	4	4
	$ADR_{j}$	-	0.1053	0.0275	0.0154			

**Table 6:** Numerical values obtained using different thresholding methods on structural textures: (1) the average method, (2) the MiMa method, (3) the Riddler method, (4) the Thrussel method, (5) the Otsu method, (6) the Pun method, (7) the Kapur method,(8) the Johanssen method and (9) the Kitler method.

Structural Textures	*N*	(1)	(2)	(3)	(4)	(5)	(6)	(7)	(8)	(9)
Milled surface	29	78.87	97.25	98.05	97.56	85.86	34.26	99.25	98.10	34.26
Fabric (fine, medium)	38	73.78	96.25	75.23	79.29	84.00	66.99	91.84	79.56	70.45
Fabric (Coarse)	29	56.20	86.82	60.02	61.26	60.68	53.16	83.95	61.07	59.20
Bamboo weave	19	52.12	88.56	65.76	61.54	52.59	51.88	91.11	62.76	39.55
Averages	115	65.24	92.22	74.77	74.91	70.78	51.57	91.54	75.37	50.87

N: number of the images.

**Table 7:** Yield values in defect detection.

Statistical Textures	*N*	Tsai [46]	$J^{*}$	Han [25]	$J^{*}$	EADL	$J^{*}$
Wood	19	92.75	4.00	82.17	3.75	97.81	1.05
Sandpaper surface	29	91.42	4.00	77.81	3.93	98.39	2.27
Wool	19	92.75	4.00	52.88	1.00	94.34	2.00
Painted surface	21	80.73	3.78	49.49	3.13	92.22	2.03
Cast metal	20	78.50	4.00	63.79	4.00	92.25	2.00
Averages	108	87.23	3.96	65.23	3.16	95.00	1.87
**Structural Textures**	N	**Tsai**	$J^{*}$	**Han**	$J^{*}$	**EADL**	$J^{*}$
Milled surfaces	29	65.05	4.00	72.82	1.00	97.25	1.10
Fabric (fine, medium)	38	58.20	3.95	62.21	3.45	96.25	1.74
Fabric (Coarse)	29	73.08	4.00	51.59	3.97	86.82	2.00
Bamboo weave	19	81.73	3.80	54.51	4.00	88.56	2.00
Averages	115	69.51	3.94	60.28	3.10	92.22	1.71
